# Subcutaneous Implants of a Cholesterol-Triglyceride-Buprenorphine Suspension in Rats

**DOI:** 10.1155/2017/3102567

**Published:** 2017-04-09

**Authors:** M. Guarnieri, C. Brayton, R. Sarabia-Estrada, B. Tyler, P. McKnight, L. DeTolla

**Affiliations:** ^1^Johns Hopkins School of Medicine, Department of Neurological Surgery, Baltimore, MD, USA; ^2^Johns Hopkins School of Medicine, Department of Molecular and Comparative Pathobiology, Baltimore, MD, USA; ^3^George Mason University, Fairfax, VA, USA; ^4^University of Maryland School of Medicine, Departments of Pathology, Medicine, Epidemiology and Public Health and the Program of Comparative Medicine, Baltimore, MD, USA

## Abstract

A Target Animal Safety protocol was used to examine adverse events in male and female Fischer F344/NTac rats treated with increasing doses of a subcutaneous implant of a lipid suspension of buprenorphine. A single injection of 0.65 mg/kg afforded clinically significant blood levels of drug for 3 days. Chemistry, hematology, coagulation, and urinalysis values with 2- to 10-fold excess doses of the drug-lipid suspension were within normal limits. Histopathology findings were unremarkable. The skin and underlying tissue surrounding the drug injection were unremarkable. Approximately 25% of a cohort of rats given the excess doses of 1.3, 3.9, and 6.5 mg/kg displayed nausea-related behavior consisting of intermittent and limited excess grooming and self-gnawing. These results confirm the safety of cholesterol-triglyceride carrier systems for subcutaneous drug delivery of buprenorphine in laboratory animals and further demonstrate the utility of lipid-based carriers as scaffolds for subcutaneous, long-acting drug therapy.

## 1. Introduction

The challenge of providing long-acting drug therapy to laboratory animals has been managed by adding drugs to the feed or water supplies [1, 2]. The utility of this method decreases when the mixture may be released inadvertently to the environment, or the drug is regulated, such as controlled substance. Feed-based drugs also may have limited postsurgical applications because pain can suppress appetite. Alternative approaches have focused on long-acting drug implants made by combining a drug with biodegradable envelops composed of lipids or polymers.

Polymers have been studied as drug carriers for neurooncology [3]. Side effects generally have been modest and localized when the polymer is implanted into neural tissue [4]. Less is known about biodegradable polymers for subcutaneous (SC) delivery of chemotherapy. Moderate to severe inflammatory reactions have been reported for SC implants of polymer-opiate constructs [5–10].

We investigated the properties of lipid-based delivery vehicles. Cholesterol, triglycerides, and phospholipids have been widely used as drug-carriers [11]. Kent described an implantable cholesterol matrix that delivered large molecules such as insulin and growth hormone [12]. Grant and coworkers demonstrated that a phospholipid-morphine liposome had prolonged activity and greater safety in mice than the free drug [13]. Liposomal strategies have been refined for the delivery of several opiates [14]. Pontani and Misra described a cholesterol-triglyceride matrix for the long-term delivery of drugs to treat chronic pain and opiate addiction [15]. The cholesterol-triglyceride vehicle appeared to provide a promising carrier to examine the delivery of antibiotics, anti-inflammatory drugs, and analgesics in surgically treated animals. To investigate the safety of this system we chose buprenorphine as a model drug. It has a high therapeutic index [16] and is a front-line analgesic for animals [17, 18].

The present report describes bioequivalence studies demonstrating that a 0.65 mg/kg dose of a lipid-buprenorphine suspension provides blood concentrations of drug greater than 0.7 ng/mL for 2-3 days. This concentration has been associated with clinically effective analgesia in mice, dogs, and humans [19–23]. A standard analgesiometric test confirmed the efficacy of the intended 0.65 mg/kg dose. Safety studies used a Target Animal Safety (TAS) trial format [24, 25]. Surgically treated male and female rats were injected with multiple overdoses of the drug suspension and monitored for adverse events (AE). The trials included clinical tests, histopathology studies, and clinical observations. The results described in the present report, when taken together with a previous report on the safety of a lipid-buprenorphine suspension in mice [26], provide further evidence that SC drug implants using lipid envelopes increase options for long-term drug therapy in rats. A preliminary account of this research has been published [27].

## 2. Methods

### 2.1. Animals and Husbandry

Studies were approved by the Johns Hopkins Institutional Animal Care and Use Committee. The protocol complies with the National Institutes of Health Guide for the Care and Use of Laboratory Animals and the requirements of the Association for the Assessment and Accreditation of Laboratory Animal Care International Program. Guidelines for TAS studies specify a minimum number of three animals per group. Four rats were used in TAS studies to account for potential morbidity during jugular vein phlebotomy. Fischer F344/NTac rats, 6–8 weeks old (male 160–180 g; female 120–130 g), were obtained from Taconic Farms (Hudson NY) and housed in an environmentally controlled room which maintained the temperatures of 20 to 26°C. Monthly health surveillance was conducted by a soiled-bedding sentinel system. Sentinel rats were considered negative for pneumonia virus of mice, reovirus, Sendai virus, lymphocytic choriomeningitis virus, rat coronavirus, sialodacryoadenitis virus, rat parvovirus, Kilham rat virus, Toolan H1 parvovirus, rat theilovirus, cilia-associated respiratory bacillus,* Pneumocystis carinii*,* Mycoplasma pulmonis,* and pinworms throughout the study.

The facility maintained a relative humidity of 30 to 70% with a 12-hour light cycle with lights on from 6 AM–6 PM. Animals were ear tagged and group housed (up to 3 per cage) during the quarantine and acclimation period based on sex. The rats were quarantined and acclimated for six days prior to dosing. No disease-related signs were noted during the quarantine/acclimation period. Prior to being placed on test, a Clinical Veterinarian approved the animals for study use. All rats appeared normal prior to dosing. The animals assigned to the two TAS trials were randomized by body weight into four groups per trial of 4 male and 4 female rats using random numbers generated by Excel (Microsoft Corp., Redmond, WA). The weight of each animal was within 10% of the mean weight of the group. On allocation and dosing, rats in the bioequivalence, efficacy, 4-day, and 12-day TAS trials were individually housed in ventilated microisolator cages throughout the study. Cages were changed daily to reduce redosing by coprophagy. Studies used soft fiber bedding from Carefresh Natural Bedding (Ferndale, WA) to house the rats. Animals were provided ad libitum with access to drinking water (Baltimore City Water System, Baltimore, MD) in disposable water bottles. The animals were provided ad libitum with access to Harlan TEKLAD Certified Global Rodent Diet 2016C (Harlan TEKLAD, Indianapolis, IN). Rats were provided with enrichment devices of polycarbonate red tubes (Bio Services, Uden, The Netherlands).

### 2.2. Experimental Design

The intended label dose of 0.65 mg/kg, which provides 2-3 days of clinically significant blood levels of drug, was established in bioequivalence trials and efficacy studies to be described using male and female rats. Single- and repeat-dose TAS trials were performed using excess amounts of the intended dose. In both safety trials, the lowest dose of drug tested was twofold greater than the intended label dose of 0.65 mg/kg. In the single-dose phase of the trials, 4 groups of 8 rats (4 male, 4 female) were dosed after surgery (described below) with 0.0 (vehicle control), 1.3 (2x), 3.9 (6x), or 6.5 (10x) mg/kg drug suspension of buprenorphine on day 0. The volumes of the vehicle control, 2x, 6x, and 10x doses were 1.0, 0.2, 0.6, and 1.0 mL, respectively. As shown in Table 1, blood and urine samples were collected at day 2. On day 4 animals were euthanized and blood and urine collected. In the repeat-dose trials, 4 groups of 8 rats (4 male, 4 female) were dosed after surgery with the vehicle control or drug suspensions containing 1.3, 3.9, or 6.5 mg/kg of buprenorphine on day 0 and following anesthesia on days 4 and 8. Surgery was not repeated on days 4 and 8. Blood and urine samples were collected at day 6. Blood, urine, and histopathology studies were conducted on tissues collected following euthanasia on day 12 (Table 1).

### 2.3. Surgery

A surgical procedure was performed to mimic the implantation of an implantable pump or a telemetry device. Each rat was anesthetized by isoflurane gas at approximately 3% with an oxygen flow rate of 1% during the procedure. The duration of the anesthesia exposure was approximately 2 minutes. Following induction of anesthesia, the scapular surface (between the shoulder blades) was shaved, washed with ethanol, and then coated with Betadine. The animal was transferred to a clean procedural area where it was assessed to ensure a deep surgical plane of anesthesia using the toe pinch method. Once deep anesthesia was verified, breathing rate and capillary fill rate were documented. Clean, sterilized forceps were used to gently grasp the skin, and then clean, sterile scissors were used to make a 4-5 mm incision through the skin only. Bone and muscle were not penetrated. The clean, sterile scissors were used to separate the skin 2 cm rostral and distal, and 2 cm lateral in the subcutis, and create a subcutaneous pocket (approximately 2 × 4 cm). The incision was then apposed and stapled using a 9 mm Autoclip® (Kent Scientific, Torrington, CT).

### 2.4. Drug Delivery

The buprenorphine-free control suspension consisted of cholesterol and glycerol tristearate (96 : 4) suspended in medium-chain triglyceride oil (8 mg/100 uL). The drug suspension consisted of buprenorphine, cholesterol, and glycerol tristearate, suspended in a medium-chain triglyceride oil (8 mg/100 uL), trade name Animalgesics for Mice. Control and drug suspensions were supplied by Animalgesic Labs (Millersville, MD). To limit stress associated with constraining conscious animals for SC injections, each rat was injected with the designated dose of test article or buprenorphine-free control suspension following surgery before they recovered from anesthesia in the single-dose trial. In the repeat-dose trial, rats were injected with drug following surgery and under anesthesia on day 0 and under anesthesia on days 4 and 8. The dose was administered SC on the middorsal area about 1 cm rostral to the surgical incision using a 25 G needle (BD, Franklin, NJ) attached to a 1 mL BD tuberculin syringe. Following dose administration, animals were transferred to a clean cage on a heating pad until recovered. Once the rat regained consciousness and demonstrated normal movement and the absence of signs of distress, it was returned to its home cage.

### 2.5. Bioavailability

Male and female rats were provided with a single dose of drug and sampled at time intervals from 8 hours to 9 days to measure blood concentrations of buprenorphine. Rat blood samples were obtained from technician-restrained, unanesthetized animals by jugular vein phlebotomy. One mL disposable syringes with 20-gauge needles were used to collect approximately 0.4 mL of blood. The sample was immediately transferred to BD tubes containing dipotassium EDTA. The samples were stored on ice for approximately 1 hour and then centrifuged to collect plasma. The plasma was stored at −20°C until it was thawed for analysis. Buprenorphine plasma concentrations were measured by the McWhorter School of Pharmacy, Samford University (Birmingham, AL), using a Shimadzu LC-20AD (Columbia, MD) and mass spec Applied Biosystems 4000 QTrap (Carlsbad, CA) assay requiring 0.25 mL of plasma. The sensitivity of the assay was 0.5 ng/mL. Mean concentration-time data was used for the pharmacokinetic analysis. Noncompartmental-analyses module in Phoenix WinNonlin version 5.3 (Princeton NJ) was used to assess the area under the curve (AUC) and the maximum plasma concentration (*C*max) time at which the* C*max is realized (*T*max). *C*max and *T*max were the observed values. The AUC was calculated by the log-linear trapezoidal rule to the end of the sample collection (AUClast) [28].

### 2.6. Efficacy

Studies were conducted with 18 F344 female rats at two dose levels: 0.65 and 1.3 mg/kg. Rats were injected with vehicle, 0.65, and 1.3 mg/kg dose of drug and tested for their tail flick response using a 55°C water bath. Tests were conducted by a female veterinarian who was blinded to the treatment group. Female rats were used because mu opioid agonists such as morphine appear to be less sensitive in female than male rats [29, 30]. The rats were injected with drug or vehicle on day 0 and examined for 5 days to monitor tail flick reaction times [31]. The rats were housed 3 per cage and cages were changed daily.

### 2.7. Clinical Observations

During the course of this study, animals were observed at the cage level once daily by a single observer prior to 9 AM for morbidity, mortality, and signs of pain or stress: abnormal breathing, tremors, ocular discharge, facial signs (squinting, eyes closed), posture, and movement and overall appearance including condition of the hair coat and grooming. Incision sites were examined at the 9 AM time for bleeding, swelling, or signs of infection. Because pica was not expected, methods to measure it, such as kaolin intake, were not used. Animals received “hands-on” detailed clinical observations once daily by a single observer after 2 PM for abnormal clinical signs (ocular discharge, motor activity, and signs of pain or distress). Incision sites were examined again at this time for bleeding, swelling, and signs of infection. Body surface was inspected for skin lesions. The process used to observe and record nausea-related behaviors has been published [27]. Briefly, observers noted in comments on the report form of hair loss and the presence of lesions as evidence of excessive grooming or self-gnawing behavior. They did not grade the amount of hair loss or the degree of biting. We considered any reports of hair loss or lesions on the paws to be signs of nausea-related behavior, and we recorded the number of rats of each sex in each experimental group that showed these signs during each observation period [27].

The observers were blinded to the treatment group. Approximately 38,000 data points were recorded in the two trials. In addition, the observers could add comments to each chart. The two male observers were Certified (AALAS) Lab Animal Technologist and Technician.

### 2.8. Body Weights

Weights of individual animals were taken for randomization (prior to study start), at midpoints (day 2 and day 6) and endpoints (day 4 and day 12) of the safety trials by an observer blinded to the dose.

### 2.9. Clinical Pathology

Blood and urine samples were collected at the mid- and endpoint of the each TAS trial. Blood was collected in the morning via jugular vein puncture. Approximately 0.5 ml of blood was collected for the midpoint clinical pathology studies. The midpoint sample was transferred to a collection tube containing dipotassium EDTA. Approximately 0.5 mL of the endpoint sample was transferred to a collection tube containing dipotassium EDTA, and 1 mL was transferred to a collection tube containing sodium citrate for coagulation factor measurements. The samples were refrigerated before analyses. The hematology examination included red blood cell (RBC) count, hemoglobin, hematocrit, mean corpuscular volume, mean corpuscular hemoglobin, mean corpuscular hemoglobin concentration, platelet count, white blood cell (WBC) count, differential blood cell count, and blood smear. Clinical chemistry tests included glucose, urea nitrogen, creatinine, total protein, albumin, globulin (as calculation), total cholesterol, alanine aminotransferase, alkaline phosphatase, aspartate aminotransferase, calcium, sodium, potassium, chloride, and phosphate. In the two TAS trials, coagulation factor measurements were prothrombin time, activated partial thromboplastin time, and fibrinogen. Because of the amount of blood needed, coagulation factors were analyzed only on endpoint days 4 and 12. Expressed urine samples were collected in the afternoon on a clean surface and pipetted into a sterile Eppendorf tube. Urine dip sticks (Bayer Multistix 10 SG Reagent Strips, Romeoville, IL) were read manually. These tests measured pH, appearance, color, protein, glucose, bilirubin, and blood.

### 2.10. Necropsy

After the final collection of blood and urine specimens on endpoint days 4 and 12, animals were humanely euthanized using CO_2_ inhalation, followed by a thoracotomy. Death was confirmed by cessation of heart rate. A comprehensive necropsy was then performed for each animal. Once the lungs were examined and weighed they were inflated with formalin to ensure proper fixation. Tissues were placed in an individually labeled container containing 10% neutral-buffered formalin, with the exception of testes (males) and eyes with optic nerves, which were preserved in modified Davidson's fixative. For short term studies, testes and eyes with optic nerves were transferred from the modified Davidson's solution to 70% ethanol 1-2 days following collection. The transfer was performed and documented by the histology lab. Containers were labeled with study number, date, group number, and animal number.

Organ weights included adrenal, brain, epididymis, heart, kidney, liver, lung, ovaries, spleen, testes, thyroid with parathyroid, and uterus with cervix. According to TAS protocols, histopathology studies were performed on the vehicle control animals and the animals given the highest dose of drug in the single- and repeat-dose trials, Table 1. Unless significant pathology was observed at the highest doses, slides from the two lower doses were not examined. Slides from the vehicle control animals and both doses in the long-term study were examined. Thirty-three tissues were harvested for histopathology examination of organs including the dorsal skin surrounding the injection site: adrenal gland, large intestine, colon, small intestine (jejunum, ileum, and duodenum), large intestine (cecum), liver, bone with bone marrow, femur, urinary bladder, lung, spinal cord with spine, brain (cerebrum, midbrain, cerebellum, and medulla/pons), lymph nodes including submandibular superficial cervical collected with salivary glands from the neck, mesenteric and pancreaticoduodenal collected with mesentery and pancreas, spleen, epididymis (males), mammary glands (females), stomach, eyes with optic nerve, ovaries (females), ventral skin, gall bladder, pancreas, heart, and parathyroid gland. Parathyroid glands were evaluated when present in the plane of section of the thyroid gland, thyroid (with parathyroid), testis (male), kidneys, and skeletal muscle (biceps femoris).

### 2.11. Statistics

A comprehensive statistical analysis (mean, standard deviations, *N*) was conducted for group mean body weight data comparing treated groups to the control group of each sex using one-way Analysis of Variance (ANOVA). An additional zero-inflated Poisson regression was conducted to estimate the dose and sex differences over time for pica behavior [32]. The zero-inflated Poisson regression model provides robust estimates and hypothesis tests for count data with a predominance of zeros. Statistical analyses (mean, standard deviations, *N*) were conducted for organ weight and clinical pathology data comparing treated groups to the control group using one-way Analysis of Variance (ANOVA). Additionally, Dunnett's *t*-test was used for control versus treated group comparisons [33].

## 3. Results

### 3.1. Bioequivalence and Efficacy Studies

The bioavailability of buprenorphine and its pharmacokinetic profile in male and female rats at defined time points following a single SC injection of the test article was examined. As shown in Table 2(a), a single dose of 0.65 mg/kg provided at least 2 days of clinically significant drug concentrations, defined as greater than 0.75 ng/mL of plasma buprenorphine. A single female had a significant concentration of plasma buprenorphine at day 7. Table 2(a) also shows the concentrations of blood present in rats dosed with 1.3 mg/kg of drug. Both male and female rats had clinically relevant plasma concentrations of buprenorphine throughout the 4 days of the single-dose and repeat-dose trials in which the lowest dose evaluated for adverse effects was 1.35 mg/kg (Table 2(a)). The estimated values for the AUC, *T*max, and *C*max are given in Table 2(b). *T*max for female rats in both dose groups was 24 hours. *T*max for male rats in the 1.3 mg/kg dose group moved to 48 hours based on a slightly greater mean blood concentration of buprenorphine on day 2. *C*max values in the 0.65 mg/kg group were 3.4 and 1.8 ng/mL for male and female rats, respectively. The estimated *C*max values for male and female rats in the 1.3 mg/kg dose group were 2-fold greater in male rats and 5-fold greater in females. The data in Table 3 shows that the extended release preparation of buprenorphine provided significant analgesic effects (*P* > 0.05) at 0.65 and 1.3 mg/kg dose.

### 3.2. Clinical Observations

All rats survived to the scheduled termination date in both trials. Rats dosed with the test article on average appeared with slightly slower movement scores when compared with the vehicle control rats on study day 0, approximately 5 hours after dose administration. Minor wounds on the front paw or wrists associated with excess grooming and self-gnawing were noted in the drug treated animals in both trials. Excessive grooming and self-gnawing behavior were not observed during the morning observation period that was given to each animal. This behavior was inferred by the absence of hair and the presence of a wound on the paw. The observer noted the findings as a comment on the animal's chart. The amount of hair loss and the degree of biting were not graded. There was no evidence of an open wound. These nausea-related signs were seen in the PM observation cycle when the animals were physically handled during an examination of the surgery site and monitoring of the entire skin surface for lesions.

Signs of nausea-related behavior were first noted in one male animal 1 day after an analgesic injection of 3.9 mg/kg, in the single-dose trial, Table 4. A single male rat in the 1.3 mg/kg dose group exhibited the behavior on day 2 and on day 3. The rats in the 3.9 mg/kg dose group showed an increasing incidence over time of the behavior and had the highest cumulative number of animals exhibiting the signs with *n* = 1 on day 1, *n* = 2 on day 2, and *n* = 3 on day 3. The highest dose group of 6.5 (10x) mg/kg exhibited a delayed onset and a lower incidence of the behavior compared to the 3.9 mg/kg (6x) group (*n* = 2 on day 3). A similar pattern was seen in the repeat-dose trial. Overall, by day 3, self-licking or paw biting was seen in 6 (25%) of the animals in the drug treated groups (beta = 1.01, SE = 0.41, and *P* = 0.01). The behavior consistently focused on the forepaws. Male and female rats exhibited similar rates of these types of behavior (beta = −0.406, SE = 0.65, and *P* = 0.53). Three of the animals in the vehicle control group of the repeat-dose trial exhibited this behavior. No signs of pain or distress were noted in the animals.

### 3.3. Weight Changes

All males and one female rat treated with 3.9 mg/kg buprenorphine lost weight between days 0 and 4 in the single-dose trial (Table 5). Two of the male rats treated with 3.9 mg/kg of buprenorphine continued to lose weight during the course of the study, while the other two gained weight between days 2 and 4 and had an overall weight gain during the course of the study. All female rats treated with 3.9 mg/kg buprenorphine lost weight between days 2 and 4, but only two of them had an overall weight loss during the course of the study. All male rats treated with 6.5 mg/kg buprenorphine lost weight between days 0 and 2, while all females treated with the same dosage gained weight. Alternatively, all female rats treated with 6.5 mg/kg buprenorphine lost weight between days 2 and 4, while all males treated with the same dosage gained weight. As shown in Table 5, a similar pattern of weight changes was seen in the repeat-dose trial. Male rats in the 6.5 mg/kg dose group treated with 3 doses in 8 days gained the least amount of weight by day 12. Female rats in the 1.3 mg/kg repeat-dose group showed the least weight gain. Overall, there were no significant changes in body weights between the vehicle control and drug treated rats.

There were no significant changes in organ weight measurements with increasing doses of drug in either male or female rats in the single-dose trial. Organ weights for livers of males in the 3.9 mg/kg and 6.5 mg/kg groups in the repeat-dose trial showed significant post hoc differences when compared to the vehicle control group, but no treatment-related effects were seen upon microscopic examination of the tissues. Organ weight measurements for brain, heart, kidneys, spleen, and thyroid remained essentially unchanged. Increasing doses of drug also had no effects on the weights for epididymis and testes in males and uterus in females. The average weight of adrenal glands in male rats increased from a control value of 0.050 to 0.074 g in the highest dose group. The change was not seen in female rats. Average liver weights decreased in drug treated male and female rats. There were no significant weight changes in the organs, other than the liver, of the rats in the long-term study.

### 3.4. Clinical Laboratory Studies

Semiquantitative (dipstick) tests of expressed urine in the single-dose trial detected urine protein in 11 (34%) and 16 (50%) of 32 rats on day 2 and day 4, respectively. In the repeat-dose trial, protein was detected in all animals in both day 6 and day 12 samples. The finding did not correlate with the dose group or sex. In the single-dose trial trace amounts of blood were detected in 21 (66%) and 10 (31%) rats on day 2 and day 4, respectively. In the repeat-dose trial, blood was detected in 7 (22%) rats on day 6 and 2 (6%) rats on day 12. In both trials, values varied from trace to moderate levels. The findings did not correlate with sex or dose group. Tests for glucose and bilirubin were negative for all samples. Appearance, pH, and color were normal in all samples in both trials.

Coagulation factor tests were performed on blood from day 4 of the single-dose trial and day 12 from the repeat-dose trial. Prothrombin time, activated partial thromboplastin time, and fibrinogen levels were normal in all dose groups in male and female rats.

Average differences in values between control and drug groups were noted in 9 of 14 hematology values and 14 of 16 clinical chemistry parameters in one or both TAS trials. These parameters were examined to determine whether changes with drug treatment varied or increased with increasing dose in male or female rats. In numerous cases, differences between control values and values from animals seen at 1.3 mg/kg dose levels were not seen in the 3.9 and 6.5 mg/kg dose levels. When differences were noted between the controls and the animals receiving drug-challenges, the changing values remained within the normal range or equaled values in the control group.

RBC values consistently decreased in day 4 and day 12 endpoint collections compared to the day 2 and day 6 midpoint values in all groups. This change was attributed to blood loss due to the previous blood collection. There was a slight increase in WBC counts after phlebotomy, an increase that we have observed in mouse phlebotomy [34]. Among the RBC indices there were no significant differences between the control groups and the animals in the 6.5 mg/kg dose groups. This indicates that the differences noted per group and sexes were random. There was no evidence of leucopenia or cytosis. Absolute values for nucleated WBCs were unremarkable, including neutrophils, eosinophils, and basophils.

Alanine aminotransferase and aspartate aminotransferase are sensitive yet modestly specific indicators of hepatocyte damage. Elevations in these serum or plasma enzyme activities are expected in drug-induced hepatotoxicity. In the present study, the enzyme levels in the drug groups closely resembled control values, even at the highest levels of drug tested. In several groups, they were modestly decreased. Serum alkaline phosphatase (ALP) can be altered by physiologic or pathologic changes in various tissues including kidney, hepatobiliary, intestine, and bone. In the present study, ALP values decrease significantly with increasing dose challenges. Sustained decreased levels of ALP have been associated with loss of appetite and fasting.

Cholesterol, BUN, creatinine, and calcium levels show small but inconsistent, and not significant, changes between the drug and control groups. The values in the drug and control groups remain within established laboratory normal values. Electrolyte levels were unremarkable: chloride, sodium, and potassium. As shown in Table 6, total protein levels on average were decreased in the highest dose group compared to controls. The decrease was not significant in the 1.3 and 3.9 mg/kg dose groups. The decrease in total protein levels appeared entirely related to decreased albumin levels with increasing drug exposure, Table 6. Primary factors affecting albumin synthesis include protein and amino acid nutrition, colloidal osmotic pressure, the action of certain hormones, and disease states. Fasting or a protein-deficient diets cause a decrease in albumin synthesis as long as the deficiency state is maintained. In the long-term study BUN values were decreased in the 1.3 mg/kg group compared to the 0.65 mg/kg group and the vehicle controls, 13.3 ± 1.0, 15.5 ± 1.0 and 16.5 ± 1.3 mg/dL, respectively.

### 3.5. Histopathology

The single-dose study reported a macroscopic observation of the thymus of one male rat in the control dose group presenting as “discolored red.” Microscopically, this presented with hemorrhage and was considered an incidental finding, possibly related to terminal cardiocentesis. No other microscopic changes were observed.

In the repeat-dose trial, macroscopic observations during necropsy included reddened mandibular lymph node in one vehicle control female and one male rat treated with 6.5 mg/kg, subcutaneous hemorrhage below the injection site in one female and two males treated with 6.5 mg/kg, fluid filled uterus in one female treated with 1.3 mg/kg, thickened skin lateral to the site of injection in one female treated with 6.5 mg/kg, and 8 × 5 × 4 mm nodule on the median lobe of the liver in one female in the 6.5 mg/kg dose group. Although organ weights for livers of males in the 3.9 mg/kg and 6.5 mg/kg groups in the repeat-dose trial showed significant post hoc differences when compared to the vehicle control group, no treatment-related effects were seen upon microscopic examination of the tissues. Inflammatory changes (granulocytic infiltration and mixed cell infiltrates) and hemorrhage were commonly seen at increased severity at or near the dorsal skin injection sites in rats in the 6.5 mg/kg dose group. Similar changes were seen in the vehicle control group. No other microscopic changes were observed.

## 4. Discussion

The objective of this study was to evaluate the safety of a lipid suspension of buprenorphine for delivery of postprocedural pain relief in F344 rats. Blood level measurements demonstrated that a single 0.65 mg/kg SC dose of a cholesterol-triglyceride buprenorphine suspension provided significant blood concentrations of drug for at least two days (Table 2(a)). Following declining mean plasma concentrations of drug on days 3 and 4, a single female rat in this dose group had an elevated blood concentration at day 7. This secondary peak may be attributed to redosing by coprophagy. Studies have shown that more than 75% of an initial dose of buprenorphine is excreted unmetabolized within one week [35]. *T*max for the intended dose of 0.65 mg/kg was 24 hours in male and female rats. The estimated AUC for the female rats given single 0.65 mg/kg was about 60% the value for male rats given the same dose but slightly greater than males when given the 1.3 mg/kg dose (Table 2(b)). A comparison of the parameters between the two dose groups is difficult because little is known about the pharmacokinetics of SC lipid drug delivery systems. Blood was not collected after day 4 from the rats in the 1.3 mg/kg test group to limit potential morbidity associated with jugular phlebotomy.

Efficacy studies using a potentially painful stimulus confirmed that a 0.65 mg/kg dose in male and female rats provided analgesia for at least three days (Table 4). Reviews of the specificity of these tests have demonstrated that thermal sensitivity tests provide a good predictor of clinical efficacy in humans [17, 36]. High thermal latency measurements at days 4 and 5 at the 0.65 mg/kg dose level are somewhat surprising because bioequivalence tests on a separate cohort of male and female rats at this dose level (Table 3) demonstrated that mean blood levels of buprenorphine dropped below 0.4 mg/mL by day 4. Yet, the results are consistent with the studies of an extended release buprenorphine depot in human volunteers showing blood level decreases of drug below 0.75 mg/ml at the end of the first week and significant clinical effects maintained for almost 6 weeks [37]. Buprenorphine and its metabolite norbuprenorphine are rapidly converted to glucuronide conjugates in rats [38]. Both conjugates have biologic activity [39] and may be relatively undetected in standard LC/MS assays.

A standard safety trial format in the present study used excess dose challenges to monitor adverse effects that might occur in real world situations where the animal was given a repeat dose of the drug or had morbidities that could enhance drug toxicity. Opiates as a class have been associated with respiratory deficiency. Studies in rats have demonstrated that compared to morphine, fentanyl, and methadone there is a ceiling effect on the action of buprenorphine [40, 41]. The present study demonstrates that prolonged buprenorphine therapy in a lipid envelop can be safely tolerated in young adult F344 rats, but the effects on other species, older animals, and transgenic rats remain unknown.

Decreasing efficacy, tolerance, and hyperalgesia have been attributed to opiates including buprenorphine [42, 43]. Studies have illustrated a complex association between experimental designs, chronic drug therapy, and hyperalgesia [44]. No significant signs of locomotor activity or hyperalgesia were observed in the studies described here. Of interest, questions concerning the clinical significance of “hyperalgesia” appear to have been mooted by the studies of chronic buprenorphine therapy using transdermal skin patches. In all cases, reported hyperalgesic signs have been minimal in rats and humans [45–48].

In 1977, Cowan et al. described the first report of buprenorphine-induced “nausea” in rats: increased stereotyped licking and biting movements [49]. Mitchell and coworkers demonstrated the association of nausea with pica by spinning rats to induce motion sickness [50]. Yamamoto et al. demonstrated that radiation sickness induced pica [51]. Takeda and coworkers confirmed the association by treating rats with opiate inhibitors to prevent nausea [52]. De Jonghe et al. demonstrated that pica in rats is an adaptive response to nausea [53]. Drugs that block mu receptors such as methylnaltrexone can be used to block the emetic effects of opiates in humans and pica in rats [54].

The two TAS trials at 1.3, 3.9, and 6.5 mg/kg doses, which were conducted on soft bedding, reduced a risk of intestinal blockage and allowed a prospective determination of the rate of emetic behavior. As shown in Table 5, the observed cumulative rate of nausea signs in the 4-day, single-dose trial increased to 25%. The observed cumulative rate was 19% in the 12-day repeat-dose trial. The animals were identified by the observers in the PM observation cycle who examined the dorsal skin surfaces of the paws. The actual rate in the 4-day trial may have been higher because animals were removed from the study before the PM observation. The rate did not increase in the 12-day trial with increasing doses of drug. In both trials, the behavior was self-limiting and produced no apparent lasting consequences. This incidence is similar to the incidence of nausea-related behavior reported in human patients treated with opiate therapies [55].

Weight loss has been cited as a deterrent to the use of postsurgical buprenorphine analgesia, and it has been linked to significant morbidity secondary to gastrointestinal blockage associated with hardwood bedding [56, 57]. A number of reports between 2000 and 2010 described weight loss in rats treated with buprenorphine without reference to the bedding used in the experiment [58], or they report using hardwood bedding without reference to previous reports associating hardwood bedding with pica [39]. Previous studies have demonstrated that the risk of pica-related gastric distress can be controlled by the appropriate choice of bedding [59]. The studies reported here confirm this observation.

## 5. Conclusion

There do not appear to be clinically significant treatment-related effects following repeated subcutaneous injections of an extended release lipid suspension of buprenorphine at 1.3 mg/kg, 3.9 mg/kg, or 6.5 mg/kg dose. Although several clinical pathology findings exceeded normal limits and urinalysis results showed abnormal parameters, there were no correlated changes or findings in body weights, clinical observations, organ weights, or microscopic evaluation of tissues.

## Figures and Tables

**Table:** (a) TAS Trial 1: single dose

Trial days	1	2	3	4
Anesthesia	+			
Surgery	+			
Drug or negative control dose	+			
Hematology		+		+
Chemistry		+		+
Urinalysis		+		+
Weight		+		+
Observed AM		+	+	+
Observed PM	+	+	+	
Histopathology				+

**Table:** (b) TAS Trial 2: repeat dose

Trial days	1	2-3	4	5	6	7	8	9–11	12
Anesthesia	+		+				+		
Surgery	+								
Drug or negative control dose	+		+				+		
Hematology					+				+
Chemistry					+				+
Urinalysis					+				+
Weight			+		+		+		+
Observed AM		+	+	+	+	+	+	+	+
Observed PM	+	+	+	+	+	+	+	+	
Histopathology									+

**(a):** 

Dose (*n* = 3/sex)	0 Hrs		6 hrs (day 0)	24 hrs (day 1)	48 hrs (day 2)	72 hrs (day 3)	96 hrs (day 4)	168 hrs (day 7)	216 hrs (day 9)
0.65 mg/kg	♂	0	1.9 ± 1.2^*∗*^	3.4 ± 0.8^*∗*^	1.9 ± 1.2^*∗*^	0.6 ± 0.2	0.4 ± 0.1	0	0
	♀	0	1.2 ± 0.3^*∗*^	1.8 ± 0.8^*∗*^	1.2 ± 0.3^*∗*^	0.7 ± 0.1	0.3 ± 0	2.3 ± 3^*∗*^	0

1.35 mg/kg	♂	0	1.9 ± 0.8^*∗*^	5.4 ± 2.1^*∗*^	6.6 ± 2.4^*∗*^	5.1 ± 2.1^*∗*^	3.5 ± 1.2^*∗*^	No test	No test
	♀	0	4.9 ± 3.0^*∗*^	9.6 ± 3.3^*∗*^	7.0 ± 1.5^*∗*^	7.4 ± 5.3^*∗*^	1.6 ± 0.6^*∗*^		

Asterisk signifies clinically significant drug concentrations, defined as greater than 0.75 ng/mL of plasma buprenorphine.

**(b):** 

Dose	Sex	AUClast hr*∗*ng/mL	*C*max ng/mL	*T*max hr
0.65 mg/kg	Male	154.2	3.4	24
	Female	99.7	1.8	24

1.35 mg/kg	Male	459	6.6	48
	Female	517.2	9.6	24

**Table 3:** Mean effects of drug on tail flick measurements at 55°C in female rats.

Dose	Time (days)				
	1	2	3	4	5
	Thermal latency in seconds				
Control	4.7 ± 2.0	6.5 ± 1.2	7.8 ± 1.5	7.1 ± 0.4	6.0 ± 0.9
0.65 mg/kg	16 ± 2.0^*∗*^	27 ± 7.6^*∗*^	24 ± 2.3^*∗*^	22 ± 6.3^*∗*^	19.4 ± 2.9^*∗*^
1.3 mg/kg	13 ± 6.8^*∗*^	26 ± 6.0^*∗*^	27 ± 14^*∗*^	25 ± 7.4^*∗*^	17.6 ± 4.1^*∗*^

^**∗**^ *P* > 0.05 for drug versus vehicle group, *n* = 6.

**Table 4:** Signs of nausea-related behavior in single- and repeat-dose trials.

Group 4 ♀, 4 ♀	Observation of biting or self-licking											
	Day											
	1	2	3	4	5	6	7	8	9	10	11	12
*Single dose*												
0.0				**∗**								
1.3		1 ♀	1 ♀	**∗**								
3.9	1 ♂	1 ♂ 1 ♀	1 ♂ 2 ♀	**∗**								
6.5			1 ♂ 1 ♀	**∗**								
*Repeat doses*				*2nd dose*				*3rd dose*				
0.0							1 ♂			2 ♂		**∗**
1.3	1 ♂	1 ♂	1 ♂	2 ♂ 1 ♀	2 ♂ 1 ♀	2 ♂	2 ♂ 1 ♀	1 ♂ 1 ♀	1 ♂	2 ♂ 1 ♀	2 ♂	**∗**
3.9	1 ♀	1 ♀	1 ♀		1 ♀	4 ♂		1 ♂	1 ♂			**∗**
6.5					1 ♀		1 ♂ 1 ♀		2 ♂ 1 ♀			**∗**
*Cumulative %*	5	8	25	9	16	19	19	9	16	13	6	

^*∗*^No observation.

**Table 5:** Mean Body Weight Gains (BWG) for male and female rats in repeat dose trial.

Dose mg/kg	BWG Day 0–4	BWG Day 4–6	BWG Day 6–8	BWG Day 8–12	BWG Day 0–12
Male					
*Control*					
Mean	10.9	4.2	−4.1	10.9	22.0
SD	1.5	1.8	6.1	0.9	6.1
*1.3*					
Mean	2.5	6.9	−1.3	11.9	20.1
SD	1.5	2.5	3.9	5.6	7.3
*3.9*					
Mean	0.3	7.1	−1.1	13.2	19.6
SD	2.4	1.4	2.5	2.0	3.0
*6.5*					
Mean	−0.7	5.6	−2.7	8.6	11.0
SD	1.5	1.9	4.2	3.9	7.5

Female					
*Control*					
Mean	2.1	2.2	−1.2	6.4	9.6
SD	2.1	2.4	3.0	1.2	2.0
*1.3*					
Mean	−1.9	2.0	−3.4	6.7	3.4
SD	0.3	0.9	3.5	1.4	5.1
*3.9*					
Mean	−3.2	3.8	−2.7	6.7	4.6
SD	1.5	2.6	1.2	5.4	6.3
*6.5*					
Mean	−1.5	3.4	−1.8	7.0	7.0
SD	3.5	1.9	2.7	5.8	6.2

**Table 6:** Decreasing plasma protein levels (mean ± SD) in combined male and female groups with increasing doses of drug.

Day	Dose(s)	Buprenorphine dose group			
		Control	1.3 mg/kg	3.9 mg/kg	6.5 mg/kg
*Total protein g/dL*					
2	1	5.9 ± 0.3	5.9 ± 0.2	5.7 ± 0.3	5.4 ± 0.4
4	1	5.7 ± 0.3	5.6 ± 0.1	5.6 ± 0.4	5.5 ± 0.2
6	2	5.8 ± 0.4	5.6 ± 0.2	5.5 ± 0.3	5.4 ± 0.2
12	3	5.8 ± 0.4	5.6 ± 0.5	5.5 ± 0.4	5.6 ± 0.1

*Albumin g/dL*					
2	1	3.2 ± 0.2	3.1 ± 0.2	2.9 ± 0.2	2.8 ± 0.2
4	1	3.0 ± 0.2	3.0 ± 0.1	3.0 ± 0.2	2.9 ± 0.1
6	2	3.2 ± 0.3	3.1 ± 0.1	2.8 ± 0.2	2.7 ± 0.1
12	3	3.2 ± 0.2	3.2 ± 0.3	2.95 ± 0.2	2.8 ± 0.1
